# The effects of an afterschool STEM program on students’ motivation and engagement

**DOI:** 10.1186/s40594-017-0065-4

**Published:** 2017-06-12

**Authors:** Jessica R. Chittum, Brett D. Jones, Sehmuz Akalin, Ásta B. Schram

**Affiliations:** 1 Department of Elementary Education and Middle Grades Education, East Carolina University, 154 Speight Building (Mail Stop 504), Greenville, NC 27858 USA; 2 School of Education (MC 0313), Virginia Tech, Blacksburg, VA 24061 USA; 3 School of Health Sciences, University of Iceland, Læknagarður/Lg-302, Reykjavík, Iceland

**Keywords:** Motivation, STEM education, Afterschool program, Mixed methods research

## Abstract

**Background:**

One significant factor in facilitating students’ career intentions and persistence in STEM (science, technology, engineering, and mathematics) fields is targeting their interests and motivation before eighth grade. To reach students at this critical stage, a design-based afterschool STEM program, titled Studio STEM, was implemented to foster motivation and engagement in STEM topics and activities. The purpose of this study is twofold: (a) to investigate how Studio STEM affected students’ beliefs about science and whether these beliefs differed from their peers who did not participate in the program, and (b) to examine a case study of one Studio STEM implementation to investigate elements of the curriculum that motivated students to engage in the program.

**Results:**

After completing two Studio STEM programs, participants’ ratings of their values for science and science competence were higher than those of non-participants. In addition, the Studio STEM participants’ motivational beliefs about science and intentions to pursue a college degree were more resilient over time than their peers. We also found that students could be motivated in a voluntary afterschool program (Studio STEM) in which they grappled with STEM concepts and activities, and could verbalize specific program elements that motivated them.

**Conclusions:**

Through this study, we found that students could be motivated in Studio STEM and that the experience had a positive impact on their perceptions about science as a field. Importantly, Studio STEM appeared to halt the decline in these students’ motivational beliefs about science that typically occurs during the middle school years, indicating that afterschool programs can be one way to help students maintain their motivation in science. Studying the program features that the students found motivating may help educators to make connections between research and theory, and their classroom instruction to motivate their students.

## Background

Increasing the number of scientists and engineers in the USA is important for meeting the demand for such professionals (President’s Council of Advisors on Science and Technology [PCAST], 2010). Although students’ interest in science often declines as they progress through school (Osborne, 2003; Simpson & Oliver, 1990), the instruction and education environments they experience can positively impact their motivational beliefs and long-term persistence (Fortus & Vedder-Weiss, 2014; Vedder-Weiss & Fortus, 2010). One significant factor in facilitating students’ career intentions and persistence in science, technology, engineering, and mathematics (STEM) fields is targeting their interests and motivation before the eighth grade (Maltese & Tai, 2010; PCAST, 2012; Tai, Liu, Maltese, & Fan, 2006). To reach students at this critical stage, an afterschool STEM program, titled *Studio STEM*, was implemented to foster students’ motivation and engagement in STEM topics and activities. Studio STEM used design-based activities in an afterschool program to provide instruction in STEM topics and concepts, with a focus on science and engineering. Initial studies of this program (Schnittka, Evans, Won, & Drape, 2015) and similar STEM programs (Cutucache, Luhr, Nelson, Grandgenett, & Tapprich, 2016) indicate that afterschool STEM programs can increase students’ STEM content knowledge. To add to these findings, we were interested in the extent to which these types of programs could also affect students’ motivation and engagement in STEM, and their beliefs about science.

One purpose of our study was to examine how Studio STEM affected students’ beliefs about science and whether these beliefs differed from their peers who did not participate in Studio STEM. Another purpose was to examine a case study of a particular Studio STEM implementation to investigate elements of the curriculum that motivated students to engage in the afterschool program. Understanding how students perceived specific aspects of the curriculum could help inform the design of similar curricula in the future. We focused this study on two primary research questions directly related to these two purposes:

- RQ1: To what extent do students’ motivational beliefs about science change as a result of participating in Studio STEM?
- RQ2: What aspects of the Studio STEM program affect students’ motivation to engage in the curriculum?

## Studio STEM program

Studio STEM was an afterschool and summer program provided at K-7 schools that was intended to engage middle school youth in STEM concepts and practices (Evans, Schnittka, Jones, & Brandt, 2016; Schnittka et al., 2015). Classroom teachers at the schools asked students to voluntarily participate in the program, and then the students agreed to participate with their parents’ permission. Studio STEM used an inquiry-based approach and an interdisciplinary curriculum to help middle school students learn about energy conservation. Classroom teachers and STEM undergraduate college students from a nearby university (referred to as *facilitators*) worked with students who learned skills and facts as they progressed through the process of solving a problem using a design approach. All of the problems involved a “Save the Animals” theme, which was intended to interest middle school youth by showing them how their energy-related behaviors could affect animals all over the world. For example, using electricity produced by burning coal can increase the levels of carbon dioxide in the atmosphere, which can have effects on animals (Gross, 2005). The “studio” part of Studio STEM refers to the informal learning environment that allows for creative explorations of strategies to solve the problems. This type of active inquiry approach to problem solving and design is consistent with the visions of national engineering and science organizations (American Association for the Advancement of Science, 1993; Katehi, Pearson, & Feder, 2009; National Research Council, 2013).

Explanations of the Save the Animals curricula and the associated materials are available elsewhere (http://www.auburn.edu/~cgs0013/engineering.htm; Schnittka, Bell, & Richards, 2010). However, because the curricula vary slightly across implementations, we will briefly describe the two curricula that we examined as part of the present study: the *Save the Penguins* curriculum and the *Save the Seabirds* curriculum.

### Save the Penguins curriculum

In the Save the Penguins curriculum, students were faced with the problem of saving penguins by designing better-insulated houses to reduce the carbon dioxide emissions that are a result of heating homes with fossil fuels. Students began by learning about thermal energy transfer through radiation, convection, and conduction (Schnittka et al., 2010). Students tested the thermal energy transfer of several different materials. Then, they used this knowledge to work in small groups to design, build, and test penguin houses made out of different materials (e.g., felt, shiny Mylar, aluminum foil) to protect a penguin-shaped ice cube from melting in the warm temperatures of a test oven. Students then shared their findings with other groups of students and used their new knowledge to redesign their penguin dwellings. The most effective dwelling design could be determined by weighing the ice penguins after a certain amount of time in the hot test oven. The group whose penguin weighed the most was the “winner” because they created a dwelling that protected the penguin most effectively in the oven.

### Save the Seabirds curriculum

In the Save the Seabirds curriculum, students were faced with the problem of reducing dependence on petroleum, which can spill and harm seabirds. Students learn about force, motion, and the Law of Conservation of Energy and then use these principles as they design, build, test, and re-test a mini solar-powered car. The car was designed to pull as much weight as possible and groups competed to determine which car design pulled the most weight. Students could test different solar panels, motors, and methods for transferring motion from motor to wheels.

## Conceptual frameworks

### Expectancy-value theory

Our first research question examined the extent to which students’ motivational beliefs about science changed as a result of participating in Studio STEM. When selecting motivation-related constructs to include in our study, we used expectancy-value theory (Eccles et al., 1983) because it was developed “to help explain gender differences in mathematics expectancies and values and how these influence boys and girls’ choices of mathematics courses and majors” (Wigfield, Tonks, & Klauda, 2009, p. 56). We, too, were interested in why middle school boys and girls choose to persist in a domain, but we were interested in the domain of science as opposed to mathematics. The Eccles et al.’s (1983) expectancy-value theory has a well-established empirical and theoretical background (e.g., Eccles et al., 1983; Eccles & Wigfield, 1995; Wigfield & Eccles, 2000). According to expectancy-value theory, motivational beliefs (i.e., expectancy/competence beliefs and task values) affect students’ choices, effort, persistence, and achievement (Eccles et al., 1983; Simpkins et al., 2006; Wigfield & Eccles, 2000). Factor analyses on questionnaire items with samples of middle and high school students demonstrated that students’ expectancies and competence-related perceptions could be combined into one expectancy/competence perceptions factor (e.g., Eccles & Wigfield, 1995; Eccles, Wigfield, Harold, & Blumenfeld, 1993). As a result, we included a measure of competence in our study. Factor analysis has also demonstrated that task values could be divided into at least three factors (i.e., interest value, attainment value, and utility value; Eccles & Wigfield, 1995), and we included measures of these values in our study as well. *Interest value* is defined as the enjoyment experienced from participating in an activity or an individual’s interest in a domain, *attainment value* is the importance of doing well on a task, and *utility value* is the usefulness of a task in terms of one’s future goals (Eccles, 2005).

### The MUSIC^®^ Model of Motivation

Our second research question examined the ways in which the Studio STEM curriculum affected middle school students’ motivation and engagement in afterschool science and engineering activities. To identify motivation-related constructs important in academic settings, we chose to use the MUSIC^®^ Model of Motivation (Jones, 2009, 2015) for several reasons, but primarily because it includes five well-established motivational constructs that have been studied over several decades, including (Jones, 2015, 2016): empowerment/autonomy (Deci & Ryan, 1991, 2000), usefulness/utility value (Eccles et al., 1983), expectancy for success (Bandura, 1986; Eccles et al., 1983), interest (Hidi & Renninger, 2006), and caring (Noddings, 1992). These five constructs were of interest to us for several reasons. First, all five constructs are malleable in educational settings; that is, they have been shown to be changeable by an instructor in the learning environment (e.g., Reeve, Jang, Carrell, Jeon, & Barch, 2004; Turner et al. 2014; Wang & Eccles, 2013). Second, these five constructs have been shown to be related to several important persistence outcomes, including engagement and motivation (Wang & Eccles, 2013; Wigfield & Eccles, 2000), interest and domain identification (Jones, Ruff, & Osborne, 2015; Osborne & Jones, 2011), and career goals (Jones, Osborne, Paretti, & Matusovich, 2014). Third, these constructs cross several different theories, which did not limit us to any one particular theory and allowed us to investigate students’ motivation as a complex, multidimensional, dynamic, and context-bound phenomenon. Fourth, validated and easy-to-implement measures of these constructs exist for use with middle school students (Jones, 2016; Parkes, Jones, & Wilkins, 2017).

The name of the MUSIC model is an acronym derived from the initial sounds of the five key components: eMpowerment, Usefulness, Success, Interest, and Caring. The five key principles of the MUSIC model are that students are more motivated when they perceive that they are *empowered*, they perceive that the content or activities are *useful*, they believe that they can be *successful*, they are *interested* in the topic or activities, and they feel *cared* for by others in the learning environment (Jones, 2009, 2015). A primary aim of the MUSIC model is to organize research-based teaching strategies into a framework that classroom instructors can easily understand and apply in a practical manner.

#### Empowerment

The empowerment component of the MUSIC model refers to teaching strategies that provide students with control and autonomy by encouraging perceptions of choice, freedom, and volition (Jones, 2009). Teachers can empower students by giving them some control over their learning environment by offering meaningful choices (e.g., choices of topics and group members), by providing opportunities for students to make decisions in the learning environment (e.g., lesson pace), and by encouraging students’ opinions.

#### Usefulness

The usefulness component of the MUSIC model includes instructional strategies that encourage students to perceive that their coursework (e.g., assignments, activities) is useful for their short- or long-term goals, or in the real world (Jones, 2009). To help students understand the usefulness of the content, instructors can (a) connect content, routines, and strategies to the real world through rationales and by defining real-life implications; (b) design tasks and activities that relate to students’ long-term, goals; (c) implement experiential, hands-on learning; and/or (d) incorporate personally relevant topics (Hulleman, Durik, Schweigert, & Harackiewicz, 2008; Jones, 2009).

#### Success

The success component of the MUSIC model includes teaching strategies that help students believe that they can succeed if they put forth the appropriate effort (Jones, 2009). Teachers can support students’ success perceptions in a variety of ways, such as by providing (a) challenging but attainable tasks and learning goals; (b) clear and realistic expectations; (c) meaningful, timely, and constructive feedback; (d) opportunities to practice and master concepts; (e) activities that are divided into manageable chunks; and (f) explanations that intelligence is malleable.

#### Interest

The interest component of the MUSIC model pertains to instructional strategies that stimulate interest in the academic activity, content, or domain (Jones, 2009). Interest includes both an affective component of emotion and a cognitive component of attention (Hidi & Renninger, 2006). Teachers can stimulate students’ interest by inciting curiosity, arousing strong emotions, introducing novelty, using a variety of instructional tools and/or tasks, including social interaction, connecting content to background knowledge and prior experiences, and using humor (Bergin, 1999; Jones, 2009).

#### Caring

Instructional strategies related to the caring component of the MUSIC model are aimed at creating a learning environment in which students feel that their instructors and classmates care about their learning and general well-being (Jones, 2009). Instructors can nurture caring by: (a) supporting students’ educational goals, (b) demonstrating that they are concerned that students achieve their learning objectives and personal goals, (c) providing opportunities for positive interactions with peers, (d) carefully designing instruction to encourage student learning, and (e) making oneself available for academic support after hours (Jones, 2009).

## Methods

### Participants

We collected two convenience samples. The first sample, which was used for the quantitative analyses addressing RQ1, included both Studio STEM participants and non-participants from two rural, low-income (Title I) K-7 schools in Southwest Virginia. The schools were located in the same rural county. We collected longitudinal quantitative data from 102 fifth-, sixth-, and seventh-grade students who were not enrolled in Studio STEM (50% from each school; 58.8% female, 41.2% male; 94.1% identified as White, 2% Black/African American, 2% Native American, and 2% selected “other” for their race). We also collected longitudinal quantitative data from 19 fifth-, sixth-, and seventh-grade students who completed both the Save the Penguins curriculum and the Save the Seabirds curriculum between the years 2012 and 2013 (47.4% at one school, 52.6% at the other; 47.4% female, 52.6% male; 100% identified as White). To collect this sample, we surveyed all of the fifth-, sixth-, and seventh-grade students who were in attendance at the schools on the days that the survey was administered. We only included students in this sample who were present at both data collection points (December 2011 and May 2013), which we describe in more detail in another section.

The second sample was used for RQ2 and included students who participated in Studio STEM at one of the K-7 schools. We collected qualitative interview data and quantitative self-report data about Studio STEM from 14 students who were enrolled in one Save the Seabirds unit during the spring 2013 semester (50% from each gender; eight in fifth-grade, five in sixth-grade, and one in seventh-grade). This sample represents an 87.5% response rate from that Studio STEM group, and includes all students present during the Studio STEM session on the day of data collection.

### Participation in curriculum

The students participated in the Save the Penguins and Save the Seabirds curricula for 90 minutes after school once a week for a duration of 6 weeks; however, two groups of students participated in Save the Seabirds over 12 weeks, which afforded them more time to complete the activities. To answer RQ2, we examined how the Save the Seabirds curriculum affected students’ motivation and engagement. Therefore, we provide more details about the specific implementation of this curriculum in the following outline of the topics and activities.

- Week 1: To provide a rationale for the program, the students watched a presentation about oil rigs and their effects on animals and the environment, and then watched a video about how solar cells work. Then, the students began working on their storyboards, which detailed their experiences and learning throughout the program.
- Week 2: The students watched and listened to a presentation on the basics of solar cells and how they work. Then, they completed a Studio STEM web-quest activity, and discussed the videos on the web-quest with their partners. Finally, in pairs, they examined real solar cells by studying their electrical energy using multi-meters, and then examined how solar cells work by studying them in parallel.
- Week 3: The students completed the multi-meter activity with their partners and then reviewed the concept of force. Then, they worked with their partners using a motor, gears, solar panels or batteries, a cup, and some string to design a device that could pull up a cup of cubes, and measured the number of cubes they could pull up at one time.
- Week 4: The students first reviewed what they learned so far in the program (energy, electricity, force, current), and then worked with their partners to finish using a motor, gears, solar panel(s), and some string to pull up a cup of cubes; however, this time they used different sized gears. When finished, they researched the word “friction” in the computer lab.
- Week 5 (data collection day): The students reviewed the previous week’s activity, the purpose of the program, and the science and engineering concepts they had learned thus far. Next, there was a presentation about friction, what it is, and what it does. Then, the teacher showed the students what happens to a toy car when the wheels are wrapped with either sand paper (high friction) or wax paper (low friction), and raced the cars on the floor of the classroom. Next, the students started designing their own solar cars with their partners. When they completed their designs, they used a lamp to see if the car moved. If it did not move, they re-designed.
- Week 6: The students continued to design and re-design their solar powered cars, and tested their cars’ movement with handheld lamps.

### Data sources

We triangulated qualitative and quantitative data regarding students’ beliefs about Studio STEM to develop a deeper understanding of their motivation and engagement. In addition, we collected and analyzed data regarding Studio STEM students’ beliefs about science in general. We posit that our sample size, although limited, is sufficient due to our concentration on qualitative methods (Glaser & Strauss, 1967). In this section, we describe the quantitative data we collected regarding students’ (1) beliefs about science in general (unrelated to their Studio STEM experiences) and (2) motivational beliefs about Studio STEM. We also explain the qualitative interview data we collected.

#### Quantitative measures

The questionnaire was titled generically as “Science Questionnaire” and was part of a larger study in which we investigated students’ motivation-related perceptions about their experiences in Studio STEM and current science classes, as well as their motivational beliefs about science (Evans et al., 2016; Jones et al., 2015). The questionnaire included items that measured constructs from expectancy-value theory (Eccles et al., 1983) and the MUSIC^®^ Model of Motivation (Jones, 2009, 2015). Unless otherwise indicated, the responses to the questionnaire items were rated by students on a 6-point Likert-type scale: 1 = *strongly disagree*, 2 = *disagree*, 3 = *mostly disagree*, 4 = *mostly agree*, 5 = *agree*, and 6 = *strongly agree*.

##### Science beliefs questionnaire

All fifth-, sixth-, and seventh-grade students (including Studio STEM participants and non-participants) in the two schools who were present when data were collected during school hours completed a Science Questionnaire in December 2011 and May 2013 (*N* = 121). In other words, 121 students completed the questionnaire at both time points. Any student who did not complete the questionnaire fully at both time points was not included in our analyses. This questionnaire assessed students’ intentions to attend college and their expectancy and value beliefs (Eccles et al., 1983) related to science (not about their beliefs related to Studio STEM), including: (a) science attainment value, (b) science interest value, (c) science utility value, and (d) science competence beliefs. We explain each of these constructs in this section and provide example items and reliability information for the present study in Table 1.

**Table 1:** Reliability evidence and example items from the Science Questionnaire

Scale	No. of items	*α*		Example items
		2011	2013	
Attainment value	4	.797	.836	“Doing well in science is very important to me”
Interest value	2	.829	.839	“In general, I find science to be very interesting”
Utility value	3	.780	.786	“What I learn in science applies to my life”
Competence	3	.840	.866	“How good at science are you?”

*Note.* Because college plans were measured with a single item, it is not listed in this table

We measured the students’ intentions to attend college using one item (“I plan on attending college”). Due to conceptual similarities between *attainment value* and *domain identification* (Eccles, 2005; Osborne & Jones, 2011), our measure of science attainment value consisted of a 4-item domain identification measure based on a modified version of the Devaluing scale (Schmader, Major, & Gramzow, 2001) altered by Jones, Paretti, Hein, and Knott (2010) to assess students’ identification with a domain (i.e., the extent to which one values a domain as a significant part of one’s self). Others have found this measure to be valid (Jones, Tendhar, & Paretti, 2016) and reliable for identification in math and engineering (*α* = .85 in Lesko & Corpus, 2006, and *α* = .84 and .89 in Jones et al., 2010, respectively). The definition of science interest value in this paper is similar to that used by Eccles et al. (1983) and Eccles and Wigfield (1995): “the subjective interest an individual has in a subject and the enjoyment experienced from performing an activity” (Jones et al., 2014, p. 7). We measured science interest value with two items that are related to items from Simpkins et al. (2006) and that replicate those from Jones, Wilkins, Long, and Wang (2012) who found the scale to be valid and reliable (*α* = .91). Our measure of science utility value assessed the extent to which the students perceived that science was useful for their futures and present lives, and is based on the measure Hulleman et al. (2008) found to be valid and reliable (*α* = .72). We measured science competence perceptions (i.e., perceived science ability, science self-concept) with three items that Eccles and her colleagues have often used, and that have been found to be both valid (e.g., Eccles & Wigfield, 1995; Simpkins et al., 2006) and reliable (*α* = .86 in Simpkins et al., 2006, a measure that included two items of the three; *α* = .86 in Eccles & Wigfield, 1995, a measure that included the three items as part of a five-item measure). The 6-point scale is different for each of the three items: “If you were to list all of the students in your class from worst to best in science, where would you put yourself? (1 = *one of the worst*, 6 = *one of the best*); “How good at science are you?” (1 = *not at all good*, 6 = *very good*); “How have you been doing in science this year?” (1 = *very poorly*, 6 = *very well*).

##### Studio STEM questionnaire

We measured students’ perceptions about their experiences during the six Studio STEM sessions using the middle/high school version of the MUSIC^®^ Model of Academic Motivation Inventory (MUSIC Inventory; Jones, 2016). A total of 14 students completed the questionnaire, all of whom were enrolled in a single Studio STEM program at one school in spring 2013. The middle/high school version of the MUSIC Inventory includes 18 items that have been validated with samples of upper elementary, middle, and high school students in music (Parkes et al., 2017) and science (Jones & Wilkins, 2013, 2015), and has been found to be reliable (e.g., in Parkes et al., 2017; empowerment *α* = .73, usefulness *α* = .86, success *α* = .92, interest *α* = .91, caring *α* = .92). The MUSIC Inventory measures students’ perceptions of the five MUSIC model components (empowerment/autonomy, usefulness/utility value, success/expectancy for success, interest, and caring), and we adapted it to reflect the students’ experiences in Studio STEM by replacing “science class,” for example, with “Studio STEM activities.” See Table 2 for example items and reliability evidence for the present study.

**Table 2:** Reliability evidence and example items for the MUSIC Inventory-middle/high school version and effort measure

Scale	No. of items	α	Example items
Empowerment	4	.779	“I had options in how to achieve the goals during the Studio STEM activities”
Usefulness	3	.921	“The knowledge that I gained in the Studio STEM activities is important for my future”
Success	4	.829	“I was confident that I could succeed in the Studio STEM activities”
Interest	3	.651	“The Studio STEM activities were interesting to me”
Caring	4	.696	“My Studio STEM teacher cared about how well I did in the Studio STEM activities”
Effort	4	.795	“I put a lot of effort into the Studio STEM activities”

*Note.* See Jones (2016) for the full instrument, including instructions and validity information

We measured effort using a four-item scale that assessed how much effort students perceived exerting during the Studio STEM activities. Our measure of effort is derived from the Effort-Importance scale from the Intrinsic Motivation Inventory (Plant & Ryan, 1985) and includes the same 6-point scale as the MUSIC Inventory. This measure has been found to be valid (Jones, 2010; Lim & Chapman, 2015; Vallerand et al., 1993) and reliable (Chittum & Jones, 2017, α = .87, .87, .85; Jones, 2010, *α* = .84, .84, .86, .84), and has an adequate Cronbach’s alpha value in the present study (Table 2).

#### Interviews

We completed structured interviews during the second-to-last session of the program with all students who attended the session (two students were not interviewed because they were absent; 87.5% response rate). The interviews lasted approximately 10 to 20 min each, and followed a structured protocol including two to five questions for each of the five MUSIC model components. The objective of these questions was to reveal the students’ perceptions of (or lack thereof) the MUSIC model components experienced during the Studio STEM sessions. To help students remember what they did during the program, we began each interview by showing them a listed summary of the presentations, activities, and demonstrations that occurred during each of the sessions. Then, we asked the students to think about those sessions in which they were present as they responded to our questions.

### School-wide data analysis

To investigate RQ1, “To what extent do students’ motivational beliefs about science change as a result of participating in Studio STEM?”, we specifically examined students’ motivational beliefs about science and school, including their plans to attend college, science attainment value, science interest value, utility value for science, and science competence beliefs. Included in our analyses were a group of Studio STEM students (*n* = 19) who completed the school-wide measure of their motivational beliefs about science at two time points, which provided both “before” Studio STEM (December 2011) and “after” Studio STEM (May 2013) scores. We gathered their responses to the questionnaire the semester prior to participating in the first Studio STEM program (Save the Penguins) and then their responses immediately after completing the second curriculum program (Save the Seabirds). We limited our sample to 19 by selecting only those students who participated in Studio STEM programs during the same time period at the same two schools, and in which both before and after scores relating to their overall science beliefs were available. In addition, we collected data from 102 of their peers who did not participate in Studio STEM. Although all present fifth-, sixth-, and seventh-grade students at both schools completed the Science Questionnaire each year, our sample was limited to 102 students because only those who completed the questionnaire fully at the same two time points (December 2011 and May 2013) were included in the study. For example, the seventh-grade students in December 2011 were not surveyed in May 2013 because they were eighth-grade students at that time and, thus, they attended a different school. We only surveyed students who were the Studio STEM participants’ peers in terms of grade level and presence at the schools.

We completed these analyses in two main stages: (1) we ran two independent samples *t* tests to compare the Studio STEM participants and non-participants’ responses at each time point (December 2011 and May 2013), and (2) we examined changes in the students’ perceptions over time by computing separate paired-samples *t* tests for the Studio STEM participants and again for the non-participants. We used a significance level (*α*) of .05 for all *t* tests.

### Studio STEM data analysis

To investigate RQ2, “What aspects of the Studio STEM program affect students’ motivation to engage in the curriculum?”, we examined one six-session Save the Seabirds program implemented during the spring 2013 semester at one school with interviews and questionnaires (*n* = 14, 87.5% response rate for this Studio STEM unit). We computed descriptive statistics for the questionnaire data and transcribed the audio-recorded interviews verbatim. We approached the analysis of the interview responses in four main stages. First, our interviews were structured to form five *a priori* categories related to the five MUSIC model components (five categories). Second, informed by Glaser and Strauss’s (1967) analytic procedures, two authors generated a list of codes using thematic whole text analysis. Third, they coded all excerpts per the identified codes and then compared their findings. Fourth, any differences in coding resulted in a discussion between the two authors and agreement on a final code. The inter-rater reliability was 81.2%. Fifth, a third researcher divided the codes into themes that emerged within each category.

## Results

### Science beliefs and college plans

**Fig. 1:**
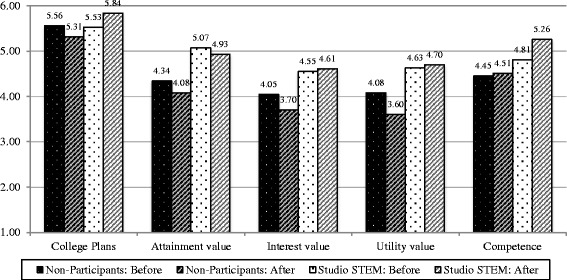
Means comparison between Studio STEM participants and the non-participants before and after program participation. Studio STEM participants *n* = 19; non-participants *n* = 102

To understand the Studio STEM students’ motivation and the effects of the program on their motivation beliefs, we examined their perceptions about the field of science. To investigate any differences between the Studio STEM students (*n* = 19) and their classmates who were not involved in the program (*n* = 102), we compared their reported science perceptions at the same two time points (December 2011 and May 2013). We first examined differences between their science beliefs at a time point prior to their involvement in Studio STEM (December 2011). Independent samples *t* tests revealed that the Studio STEM students started with significantly higher science attainment value than their peers, while all other perceptions were statistically similar (see Table 3 and Fig. 1). In May 2013, following the Save the Seabirds program, the Studio STEM participants reported significantly higher college plans, science attainment value, science interest value, science utility value, and science competence beliefs than their peers who did not participate in Studio STEM (see Table 4 and Fig. 1).

**Table 3:** Comparing Studio STEM participants and non-participants before program implementation

Variable	Group	*n*	*M*	*SD*	*t*	*df*	*p*
College plans	Studio STEM	19	5.53	1.26	−0.11	119	.916
	Non-participants	102	5.60	0.99			
Attainment value	Studio STEM	19	5.07	0.76	3.58	119	.001
	Non-participants	102	4.34	1.05			
Interest value	Studio STEM	19	4.55	1.25	1.57	119	.127
	Non-participants	102	4.05	1.45			
Utility value	Studio STEM	19	4.63	1.28	1.75	119	.094
	Non-participants	102	4.08	1.14			
Competence beliefs	Studio STEM	19	4.81	1.06	1.34	119	.193
	Non-participants	102	4.45	1.03

**Table 4:** Comparing Studio STEM participants and non-participants after program implementation

Variable	Group	*n*	*M*	*SD*	*t*	*df*	*p*
College plans	Studio STEM	19	5.84	0.50	3.07	119	.003
	Non-participants	102	5.31	1.29			
Attainment value	Studio STEM	19	4.93	0.93	3.51	119	.001
	Non-participants	102	4.08	1.20			
Interest value	Studio STEM	19	4.61	1.22	2.88	119	.007
	Non-participants	102	3.70	1.47			
Utility value	Studio STEM	19	4.70	1.07	3.93	119	< .001
	Non-participants	102	3.60	1.34			
Competence beliefs	Studio STEM	19	5.26	0.68	4.10	119	< .001
	Non-participants	102	4.51	0.99			

In addition, we examined changes in the students’ perceptions over time. We found that the non-participants’ perceptions about science attainment value (*t*_(101)_ = 2.10, *p* = .039), science interest value (*t*_(101)_ = 2.29, *p* = .024), and science utility value (*t*_(101)_ = 3.33, *p* = .001) significantly decreased over time (see Fig. 1 for mean values). The *t*-test results and analysis of the overall means indicates that the non-participants’ intentions to attend college did not significantly change (*t*_(101)_ = 1.81, *p* = .073), and only their science competence beliefs increased over time, although the change was minor (*t*_(101)_ = −0.542, *p* = .589). Conversely, we found that the Studio STEM participants’ motivational beliefs did not significantly decrease over time; in fact, unlike their peers, their reported beliefs maintained in terms of college plans (*t*_(18)_ = −1.302, *p* = .209), attainment value (*t*_(18)_ = 0.587, *p* = .564), interest value (*t*_(18)_ = −0.150, *p* = .882), and utility value (*t*_(18)_ = −0.228, *p* = .882), and, further, the Studio STEM participants reported significantly higher science competence beliefs (*t*_(18)_ = −2.22, *p* = .040) after participating in Save the Seabirds (see Fig. 1 for mean values).

### Perceptions about Studio STEM

We investigated the Studio STEM participants’ motivational beliefs related to the spring 2013 Save the Seabirds curriculum to better understand how an afterschool program such as Studio STEM can affect students’ motivation and engagement.

#### Questionnaire

The students (*n* = 14) reported that they were empowered in the program (*M* = 5.23, *SD* = 0.78), found the activities and content useful (*M* = 5.02, *SD* = 0.97), felt that they could be successful (*M* = 5.57, *SD* = 0.54), found the tasks to be interesting and enjoyable (*M* = 5.48, *SD* = 0.76), and perceived that others in Studio STEM cared about their successes and personal well-being (*M* = 5.27, *SD* = 0.83). In addition, they reported that they put forth a high amount of effort in Studio STEM (*M* = 5.50, *SD* = 0.60). Overall, these values ranged from 5.02 to 5.57 on a 6-point Likert-type scale (for which 5 = *agree* and 6 = *strongly agree*), indicating that the students were highly motivated to engage in Studio STEM.

#### Interviews

Research and theory posit that students often engage in and put forth effort during learning tasks when they perceive that they are empowered, the content is useful, they can be successful with effort, the content is interesting, and others in the learning environment are caring (Jones, 2009). Thus, we interviewed students (*n* = 14) about their perceptions of each of the five components of the MUSIC model to better understand how these perceptions influenced their motivation in Studio STEM. Based on the interview responses, we developed a total of 98 codes that we discuss in the next sections. We present our results in the order of the MUSIC model acronym. Of these 98 codes, the majority (88.1%) were descriptions of positive/motivating program features. The percentages cited in the following sections are out of the total number of coded responses (i.e., excerpts) for each category.

##### Empowerment

To study students’ perceived empowerment, we asked them to describe the choices they perceived during Studio STEM and two themes emerged from their responses: the students perceived that they (a) had some choice over how to work on the activities (86%) and (b) had choice regarding when to participate during the sessions (14%).

The activity choices the students mentioned tended to focus on their use of specific types, quantities, and sizes of materials during the activities (e.g., gears, solar panels, motors, wheels), as well as how they designed and assembled their solar powered car and the device that lifted a basket of cubes (e.g., how to assemble the gears, location of wheels, location of motors). For example, one student noted that he had choice over “how heavy or light the car is.” Another student explained that she had “a lot” of choice noting that, “We got to pick how we got to make our car, what design we wanted, where to put the motor, where to make the wheels go, which wheels we wanted. Entirely up to us.” In addition, some students explained that they had some choices regarding when and how they would participate during the presentations and activities, and when working on EDMODO, a Facebook-like social learning community application that is designed for K-12 students, teachers, and parents to interact in a secure environment.

##### Usefulness

We asked students to describe what they had done in Studio STEM that (1) was useful to their lives currently, and (2) what was useful for their futures. Three themes emerged: (a) learning useful information about specific science and engineering topics, and learning concepts that are (b) useful for school and professional success, and (c) useful for doing something outside of school. In addition, two students (7% of the coded excerpts) believed that they would not have any future need for the information they learned in Studio STEM.

Several responses (19%) described specific topics that the students considered useful for their present lives, including learning about gears, motors, solar cells, and friction. However, even though they stated that specific topics and concepts were important, they did not always explain why or how. For example, one student said, “Friction is kind of useful because in some cases you might need to know when friction is too much or too little. Maybe when you’re in science class or something, it’s just a little difficult [to know when].” At the same time, others more readily related the learning material to real world uses (e.g., “I like working with cars a lot. It’s kind of nice to know how to use the gears in a certain place where they could move faster and still pull a heavy load.”). Many responses (41%) indicated that the students believed they learned information that was either useful for their current science class, or would be useful for pursuing a career in science or engineering in the future. In particular, several females specifically expressed interest in STEM careers. One female student contested female stereotypes: “Even though boys think that girls can’t be engineers because they think they are too girly and everything, I think that girls can be engineers so this is a good way of learning how to be an engineer.” Regardless of their expressed intentions, it is difficult to know if these students’ goals were influenced by the Studio STEM curriculum or by weekly interactions with male and female undergraduate science and engineering students who volunteered as facilitators. Finally, multiple responses (33%) also indicated that topics learned in Studio STEM were useful for some tasks outside of school (now and in the future), such as repairing cars and fixing technological devices. For example, one student explained, “We’ve learned how to build cars and how they would look, and how to repair them.”

##### Success

We asked students what made them feel like they could be successful in Studio STEM, and three themes emerged: (a) others were helpful during Studio STEM, (b) they felt successful during specific activities and presentations, and (c) they already felt like they were good at or enjoyed related topics or activities. Also, six students (23% of the coded excerpts) explained that there were moments when they did not feel successful during Studio STEM, including at times when building the model car and when working with motors to lift a basket of cubes.

Some of the more common responses (35%) suggested that the support systems built in to Studio STEM were important to the students’ perceived success, including support from the facilitators (e.g., “We were all mixed up . . . but then [the teacher] came over to help us and then we . . . started fixing it and, doing what we were supposed to do, we got it right”) and other students in the program (e.g., “My partner, she always motivates me. Sometimes when she said ‘oh no it’s fine, just keep on trying’ and having that positive attitude, and we just kept on going and going until we got it.”). Other responses (31%) described certain activities and topics during which the students felt successful, including when they worked on the solar cars, solar panels, motors, pulling up a basket with cubes, and measuring voltage and amps with multi-meters. Finally, three students explained that they felt successful because they were very interested in science and held high science competence beliefs, or had been successful on similar tasks in the past. For example, when asked to describe what made him feel successful, one student responded that he already felt confident on task components: “I like building Legos. I’m good at building Legos and just working with other people.”

##### Interest

We asked the students to explain what was interesting about the presentations and activities (two questions), as well as what was boring about the presentations and activities (two follow-up questions). Five themes emerged from their responses about what was interesting: (a) the structure of the program and daily activities, (b) certain presentations and demonstrations, (c) specific hands-on activities, (d) working with others, and (e) feeling successful and learning new things during the activities. In addition, we found three themes in their responses about boring aspects: (a) when it was difficult or challenging, (b) when information was reviewed, and (c) some activities, presentations, and topics.

Responses (16%) suggested that some students found several structural aspects of Studio STEM to be interesting, such as the teaching methods, the structure of the presentations, and that they had many opportunities to experiment by themselves and participate in hands-on activities (e.g., “The [facilitators] explained a lot of things, [the presentations] were fun to watch, and [the teachers] told us about what we were doing and what we were going to do.”). In addition, they listed many things they considered to be interesting, including multiple presentations and presentation topics (19%; e.g., friction, oil rigs and their effect on the environment, light and solar cells, and the demonstration including a toy car with wheels covered in wax paper and sandpaper), and activities (31%; e.g., working with gears, designing and building solar cars, using multi-meters, using EDMODO, and working on storyboards). They also explained that working with other people was interesting (8% of responses) and, in particular, collaborating with their partners on the activities. For example, one student described the benefits of teamwork during one activity: “When we worked with our partner using the motor, it was fun because we both got to intertwine our ideas all together and see how they worked.” Finally, some responses (5%) equated interest to learning and success, and explained that it was interesting when they learned new things and felt confident in succeeding. For instance, one student described how her interest related to her sense of accomplishment and learning:

You got to build and you got to re-create, and then figure out your mistakes, and then do it again. If you did not know it [before], you thought, “oh, I get it now,” and then you are glad that you accomplished something that you have never done before.

The students also described several aspects of Studio STEM that they considered boring, with more responses concerning the presentations being boring than the activities. Two excerpts (3%) explained that sometimes it was boring when the presentations or activities were difficult or confusing, suggesting a connection between success beliefs and perceived interest (e.g., “Solar cells are kind of confusing and the solar panel things are kind of confusing, and when I get confused, I get bored.”). Inversely, five additional responses (8%) suggested that the presentations and activities were boring when the students felt like the material was reviewed too extensively (e.g., “Usually the stuff I already know [is boring] . . . and sometimes I just think, ‘I know all of this stuff, can we just go on?’”). Finally, several responses (11%) indicated that some topics and tasks were boring. Most of the responses concerned the presentations (five out of seven responses; e.g., presentations about solar cells, electricity, voltage, current, motors and engines). One student attributed the problem to the instructional methods: “Sometimes it would get a little boring because they just didn’t explain it in a very exciting way, it was just very dull.” Further, two students said that an activity was boring (working on the computers and creating a device to lift a basket).

##### Caring

We asked students to talk about both the facilitators’ caring and the other students’ caring, and seven themes emerged from their responses. Three themes pertained to the facilitators: (a) they helped the students be successful, (b) they communicated that they cared about the students and their successes, and (c) their choice to develop and participate in Studio STEM communicated caring. No students mentioned anything negative about the facilitators’ caring. Another three themes describe their perceptions about the other students’ caring: (a) partnerships between students were trusting and helpful, (b) they were helpful and wanted others to be successful, (c) they showed respect towards and interest in others’ work. In addition, one student indicated that the other students were not interested in others’ work.

Many students agreed that the facilitators were helpful (38% of the responses). They explained how the facilitators helped them when they were stuck, confused, or missed something, as well as used teaching methods that helped them to understand the content. The facilitators also asked them how they were progressing on the activities and encouraged them to keep trying. For example, one student explained, “They kept me going. If I had trouble they’d always help me a lot. They’d say, “You should try another thing.” [Once] I tried using a tire as a gear [laughs], I tried pulling it up with a tire!” The students described facilitator behaviors like making encouraging statements, being funny, and communicating that they wanted to students to persevere. The second theme pertains to how the facilitators communicated that they wanted the students to be successful in Studio STEM and cared about their personal well-being (9%). The facilitators showed that they not only wanted to help the students when they were struggling but also that they genuinely desired student success, and enjoyed their job. The third theme concerned the facilitators’ choices to participate in Studio STEM and the reasons why they were motivated to develop the program (4% of responses). The fact that Studio STEM was developed communicated caring to one student: “Well first of all, they wouldn’t even have this program if they didn’t care.” Another student thought that they cared because they chose to be facilitators in Studio STEM and to educate students.

In the first student caring theme, several responses (6%) described effective and helpful partnerships. The students explained that their partners helped each other and worked well together to solve the problems. In addition, a majority of the students’ responses about their peers’ caring (34%) indicated that others in the program were also invested in their successes. They explained that the other students helped when they struggled, such as offering specific suggestions, allowing struggling students to look at their designs, and communicating when a specific tactic did not work for them (e.g., “When we built our cars [the other students] would look at the cars and inspect them, and they would test them for themselves. We felt like they cared about how successful we were.”). Finally, responses (6%) in the third theme indicate that there were positive levels of respect and interest among the students (e.g., “They didn’t like yell at us or be mean to us. They actually respected us.”).

## Discussion

The primary objective of this study was to investigate the effects of an afterschool science and engineering program, Studio STEM, on upper-elementary and middle grades students’ beliefs about science, and their motivation and engagement in the program. Our discussion of these findings centers first on the students’ overall science beliefs, including changes noted over time, and then their motivation in Studio STEM.

### Motivational beliefs about science and college plans

Overall, our findings suggest that Studio STEM had a positive impact on the participants’ motivational beliefs about science and plans to go to college. The Studio STEM students held fairly similar perceptions to their peers before they participated in the program, with the exception of the level of importance they attributed to science as a part of their self-identities. We posit that their higher science attainment value is consistent with students who were recommended for a voluntary afterschool program by their teachers, and who agreed to participate. Following participation in two Studio STEM programs (Save the Penguins and then Save the Seabirds), the Studio STEM students maintained their increased attainment value, and, furthermore, they reported that they found science more interesting and useful, and were more competent in their science abilities than their peers. Because Studio STEM students reported these higher values than non-participants, participation in Studio STEM likely facilitated their development of these motivational beliefs.

The results also suggest that the Studio STEM participants’ motivational beliefs about science and intentions to pursue a college degree were more resilient than their peers. The non-participants’ motivational beliefs significantly declined over time, which is consistent with previous research showing that academic motivation often wanes during the upper elementary and middle grades (e.g., Eccles et al., 1993; Jacobs, Lanza, Osgood, Eccles, & Wigfield, 2002), and specifically in STEM fields (e.g., Chittum & Jones, 2017; Osborne, 2003; Simpson & Oliver, 1990). Moreover, a previous study of this specific population (*N* = 913) provided evidence of this decline in motivation beliefs over time (Chittum & Jones, 2017). However, the Studio STEM participants reported increased competence beliefs and maintained attainment, interest, and utility values, as well as college intentions. Although this study is only an initial step in examining students’ motivation in this way, our findings may indicate that participation in problem-based afterschool science and engineering programs like Studio STEM can serve in assuaging the documented decline in science-based motivation perceptions many students experience during those academic years. In turn, due to previous research (Maltese & Tai, 2010; Tai et al., 2006), we speculate that, because these students entered secondary education more motivated and engaged in STEM fields, they were more likely to pursue STEM fields in college and/or STEM-related careers.

Although this study did not allow us to identify any one particular aspect of Studio STEM that led to reducing this decline in motivation, we speculate that a combination of the features of the Studio STEM program were responsible. For example, the program empowered students in science, demonstrated the usefulness of science through activities, helped students to be successful in science, interested them in science activities, and provided a caring environment in which to participate in science. These principles are consistent with the MUSIC Model of Motivation (Jones, 2009) and with decades of research by many researchers in using different theoretical orientations (e.g., Schunk, Meece, & Pintrich, 2014; Wentzel & Wigfield, 2009). It is also consistent with evidence that using the MUSIC model components can help students to become more identified with a domain such as science (e.g., Jones et al., 2015) and persist in a specific domain. It appears that, in this case, instead of experiencing an *increase* in identification (which is similar conceptually to attainment value), students did not experience a *reduction* in identification. The program was set up to provide regular models of those who have persisted in STEM fields, including daily college student volunteers who were undergraduate STEM majors. According to social cognitive theory (Bandura, 1986), observation of and interaction with models in the environment can encourage students to follow suit by continuing their studies and persist in a domain or on a task. Of course, Studio STEM was not the only influence on these students’ science beliefs. These beliefs were also likely influenced by their regular science class and their experiences outside of school.

### Motivation in Studio STEM activities

The high mean values for each of the subscales in the Studio STEM questionnaire, and the high proportion of interview responses that described positive attributes of the program suggest that the students were motivated to engage while participating in Save the Seabirds activities. The students talked about many program features they found to be motivating. The following points are organized by MUSIC model component, rather than by level of importance.

#### Empowerment

The students mentioned several program features that they considered empowering. One of the strengths of problem- and design-based learning programs is that they are considered *student-centered* because students are often encouraged to take charge of and guide their learning (Kember, 1997). These data offer several examples of how students were able to make choices and engage in student-centered learning. The choices the students described were primarily meaningful and relevant to the overall course or specific lesson, and sometimes the program more generally, which provided them with some authentic and significant control. Previous research indicates that providing choices (Patall, Cooper, & Wynn, 2010), especially *meaningful* choices, can positively impact students’ motivation and engagement (Patall, Cooper, & Robinson, 2008). An example of a meaningful choice was when the students could control how they designed their solar-powered cars. Furthermore, the students described having *action choices*, or choices whether to engage or not to engage, which have also been found to facilitate autonomous motivation, as they communicate an even greater sense of empowerment to the students involved (Reeve, Nix, & Hamm, 2003). For example, students believed that they had a choice pertaining to when and how they would participate in EDMODO, and could choose not to participate, if preferred.

#### Usefulness

The students indicated multiple topics and program components that they considered useful both in and outside of school. Brophy (2008) explained that appreciation and value for content can be nurtured by making connections between the material and students’ lives at home, describing real-world application, and relating course content and activities to future careers and academic paths. Our data suggest that the students’ experiences were conducive to developing such values and appreciation for the science and engineering concepts emphasized in Studio STEM. Research suggests that communicating utility value is especially effective when students have increased expectancies for success (Durik, Scheter, Noh, Rozek, & Harackiewicz, 2015). Because these students’ competence perceptions were higher than their peers following their participation in the program, it is possible that such efforts to communicate utility value were particularly successful with this group.

#### Success

The interviewed students described multiple built-in program structures that supported their expectancies for success. They explained that the other students and the program facilitators were especially salient, and, further, that collaboration was an integral component to completing the activities. Prior research has shown that having ample achievement support (from peers and teachers), and teachers who hold high expectations and encourage students to seek challenges, as the students we interviewed described, can facilitate the development of positive success beliefs (e.g., Bandura, 1986; Urdan & Turner, 2005). Researchers also posit that it is especially important to provide support in ill-structured, problem-based learning environments such as this, as students are using higher-order cognitive processes to complete the activities and tasks (Kirschner, Sweller, & Clark, 2006). In addition, because these students were offered increased autonomy over their learning in Studio STEM, these strong supports were likely important to maintaining their positive success beliefs while regularly engaging in meaningful decision-making. Research suggests that students with lower success beliefs often prefer less autonomy (Könings, Brand-Gruwel, & Elen, 2012) and perform worse when given ample freedom (Tai, Sadler, & Maltese, 2007).

#### Interest

Many students agreed that activities and topics in the program sparked their interests. The students’ responses suggest that the program triggered and maintained their situational interests (Hidi & Renninger, 2006), in that they described many specific topics and activities that were “fun” to do, or were presented in an interesting fashion. Some students also described interests that more closely align with emerging individual interest, which is considered a more developed and lasting form of interest (Hidi & Renninger, 2006). This is evident in the positive affect they associated with the tasks (e.g., using terms like “enjoy,” “glad,” and “accomplishment”), their connection between enjoyment and repeated and consistent efforts to solve difficult problems, preference for exploring the problems on their own and in a hands-on manner, and intentions to exceed required task expectations by engaging deeply and collaboratively during the activities (Ainley & Ainley, 2011; Hidi & Renninger, 2006).

#### Caring

Most students agreed that the facilitators’ consistent and positive support was important in fostering a caring environment. Moreover, multiple students explained that the other students in the program offered suggestions and helped struggling peers. This caring learning environment indicates that the facilitators and students maintained a secure level of attachment. In particular, the facilitators’ behaviors that the students described are consistent with secure teacher-student relationships that communicate secure attachment and caring in the classroom (Bergin & Bergin, 2009; Noddings, 1992), such as their hard work and time spent participating in the program to help the students, in addition to freely solicited encouragement and support. The students’ interactions with one another also indicate social competence (Wentzel, 2005) and secure student-teacher relationships in Studio STEM (Bergin & Bergin, 2009), which can all be influenced by program facilitators.

#### Overall perceptions

The students generally described positive motivational experiences (88.1% of the responses). In all, the students were able to communicate many specific program elements they found to be motivating, which served to explain their highly positive responses to the questionnaire items. However, we also asked about and received their recommendations for program improvement. Of these responses, most students agreed on one point: that it was boring when information was reviewed extensively or the teacher belabored initial instruction, such as when students felt that they already knew the information well enough and were ready to move forward. Another weakness prevalent in their responses pertains to the elements of direct instruction in Studio STEM. Both of these points indicate that the presentations and instructional methods may be key areas to target for improvement, which also aligns with the results of a previous study wherein we investigated the motivation of a different Studio STEM Save the Seabirds group (Jones et al., 2015). Although direct instruction can be vital for learning, it may not facilitate motivation as well as other program elements. Nevertheless, past researchers have posited that direct instructional methods and students’ prior knowledge are key factors in their performance (Schwartz & Bransford, 1998), especially in the ill-structured learning integral to problem- and design-based learning programs (Kirschner et al., 2006; Sweller, Kirchner, & Clark, 2007). We speculate that more intentionally focusing on other MUSIC model components during the presentations may serve to alleviate some of these negative perceptions, such as by making them more interesting or by relating presented information to students’ lives and the real world.

#### Interactions among MUSIC perceptions

Multiple interview responses indicated interactive relationships between the students’ perceptions of several MUSIC model components. Some students associated interests with success beliefs. They reported that they considered activities and presentations boring when a task was considered challenging or too easy. On the other hand, others explained that they felt like they could be successful when they were interested in the activities, and also that they were interested when they felt confident and successful. Some students described that they were interested when they were empowered to experiment by themselves on the activities. Similarly, several students described Studio STEM topics that they found interesting outside of school as both useful and interesting. This is consistent with the findings of other researchers who have documented that when the utility value of the content is clear, the experience of interest and enjoyment is more likely (Ainley & Ainley, 2011). Previous research has also shown the MUSIC components are correlated yet perceived as distinct constructs in middle grades science students (Jones & Wilkins, 2013). Thus, these findings align with notions that academic motivation is a complex and dynamic phenomenon in which motivation beliefs likely interact with one another and are not linear, isolated variables (Kaplan, Katz, & Flum, 2012). In practice, these associations suggest that, by designing one’s instruction to target even one MUSIC model component, educators may in fact be supporting two or more by proxy.

## Limitations

In this section, we discuss two potential limitations of this study: (a) the sample size and (b) the selection of participants into the program. First, our sample was somewhat limited in the number of students who participated. However, we posit that our sample is sufficient due to our concentration on qualitative methods (Glaser & Strauss, 1967). For the school-wide science questionnaire (RQ1), there were 19 Studio STEM participants, which included participants in the Studio STEM program at two schools. For the Studio STEM-specific data pertaining to one Studio STEM program (RQ2), we included data from 14 participants (an 87.5% response rate from that program). Due to the small program size and inherent limitations on program enrollment, we posit that our Studio STEM participant samples were adequate and representative of the population of study. For RQ1, we limited our sample of non-participants to students who completed the science questionnaire at two specific time points. Further, the sample represents all students who were present at the school during both data collection events; thus, the sample is representative of the school population and the Studio STEM participants’ classmates.

A second limitation concerns possible selection bias in Studio STEM participation. Studio STEM program selection was based on teacher recruitment and recommendations. Furthermore, the program was voluntary in nature. Because of these procedures, it is possible that the students who participated in Studio STEM were more likely to be motivated and persist in STEM fields. We addressed this concern by running independent samples *t* tests, which compared participants’ and non-participants’ perceptions at two time points (before and after).

## Conclusions

Studio STEM largely had a positive impact on the participants’ motivation and engagement in the program-based science and engineering activities, as well as their motivational beliefs about science and intentions to pursue a college education. Although students cited some negative aspects of the program and activities, their perceived motivation and interview responses were consistently and mostly positive. Through this study, we found that students could be motivated in a voluntary afterschool program in which they grappled with STEM concepts and activities, and that the experience had a positive impact on their perceptions about science as a field. Furthermore, the students were able to verbalize specific program elements that they found to be motivating. Studying the program features that the students found motivating may help educators to make connections between scientific research and theory, and their classroom instruction to motivate their students. Finally, Studio STEM appeared to halt the decline in these students’ motivational beliefs about science that typically occurs during the middle school years. This finding indicates that afterschool programs can be one way to help students maintain their motivation in science.
